# Supplementary material for: Architecture and mechanism of the late endosomal Rab7-like Ypt7 guanine nucleotide exchange factor complex Mon1–Ccz1
Source: Nat Commun. 2017 Jan 4;8:14034. doi: 10.1038/ncomms14034 (PMC5216073; doi:10.1038/ncomms14034)
Supplement: Supplementary Information — Supplementary figures, supplementary tables and supplementary references. [file ncomms14034-s1.pdf]

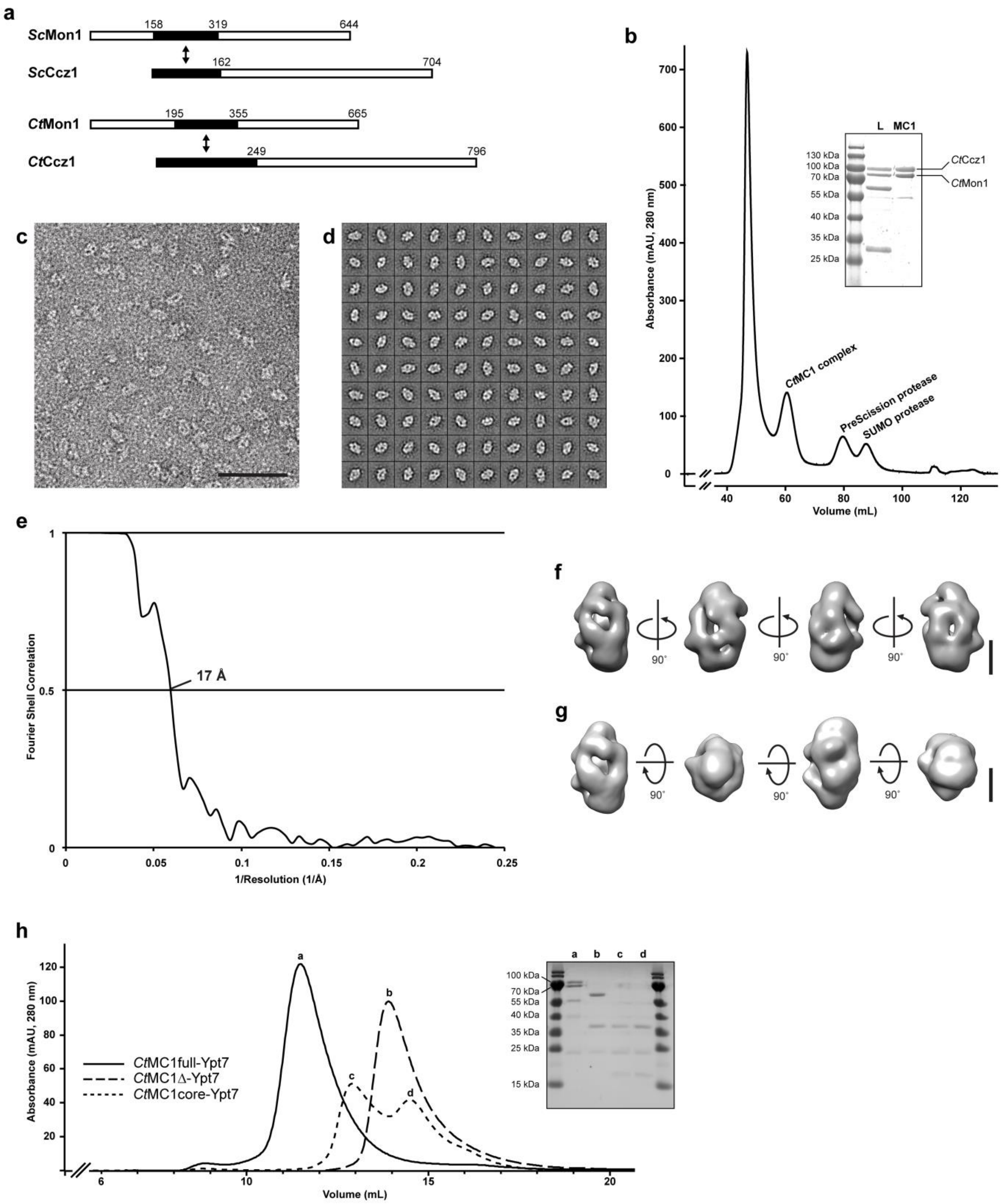
a
ScMon1
ScCcz1
CtMon1
CtCcz1
b
CtMC1 complex
PreScission protease
SUMO protease
Absorbance (mAU, 280 nm)
Volume (mL)
L
MC1
CtCcz1
CtMon1
c
d
e
Fourier Shell Correlation
17 Å
1/Resolution (1/Å)
f
g
90°
h
Absorbance (mAU, 280 nm)
Volume (mL)
CtMC1full-Ypt7
CtMC1Δ-Ypt7
CtMC1core-Ypt7

Supplementary Figure 1. Architecture of MC1. (a) Domain organization of *S. cerevisiae* and *C. thermophilum* Mon1 and Ccz1. The positions of the longin domains are marked by black bars. (b) Purification of full-length *Ct*MC1 on a Superdex 200 (16/600) gel filtration column for electron microscopy. L: load; MC1: pooled peak fractions of *Ct*MC1. (c) Micrograph of negatively stained *Ct*MC1 complex. Scale bar corresponds to 50 nm. (d) 100 class averages from 22,165 particles that were initially used as templates for *ab initio* 3D structure determination; box size is 24x24 nm) (e) Fourier Shell Correlation of the negative stain reconstruction of the full-length Mon1-Ccz1 complex. (f) Different side and (g) top views of the *Ct*MC1 3D-reconstruction rotated by 90°. Scale bar is 5 nm. (h) Analysis of MC1 and subcomplexes bound to Ypt7 on a Superdex200i (10/30) gel filtration column. *Ct*MC1full: Mon1 1-665, Ccz1 1-796; *Ct*MC1Δ: Mon1 140-665, Ccz1 1-249; *Ct*MC1core: Mon1 195-355, Ccz1 1-249. Peaks correspond to a: ~360 kDa; b: ~85 kDa; c: ~150 kDa d: ~60 kDa).

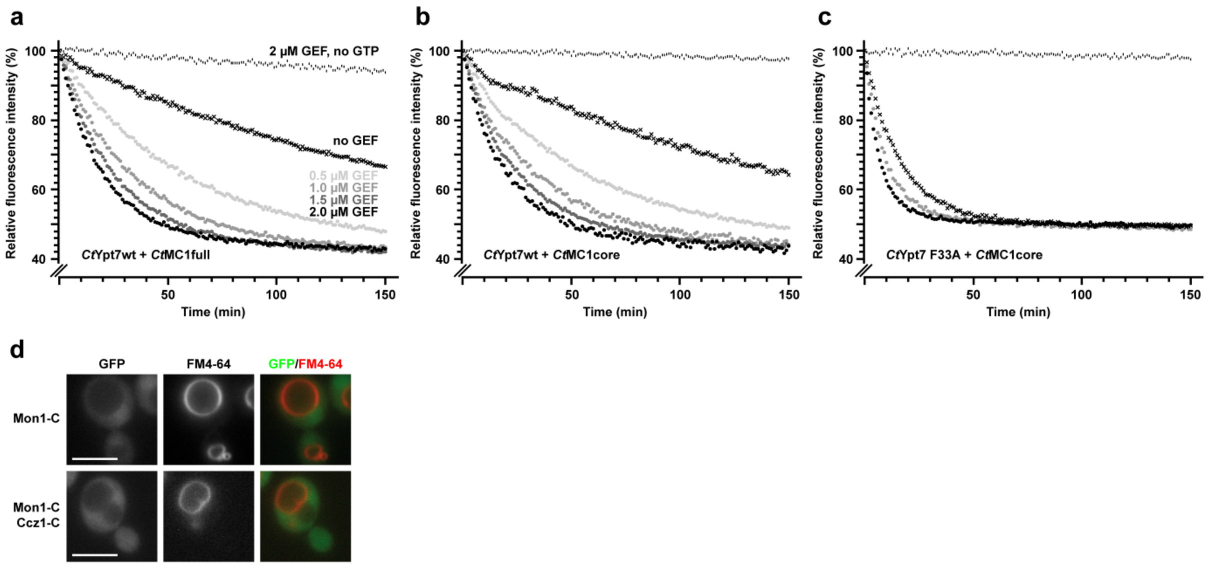

Supplementary Figure 2. Release of MANT-GDP was monitored in the presence of GTP or GEF only or both for *Ct*Ypt7 with *Ct*MC1full (a), *Ct*Ypt7 with *Ct*MC1core (b) and *Ct*Ypt7 F33A with *Ct*MC1core (c). Initial rates were determined for different GEF concentrations. (d) GFP-tagged Mon1 C-terminus (Mon1-C) and a fusion between the Mon1 and Ccz1 C-termini (Mon1-C Ccz1-C) do not localize to endosomal structures when expressed in a wild-type background. Scale bars represent 5 µm.

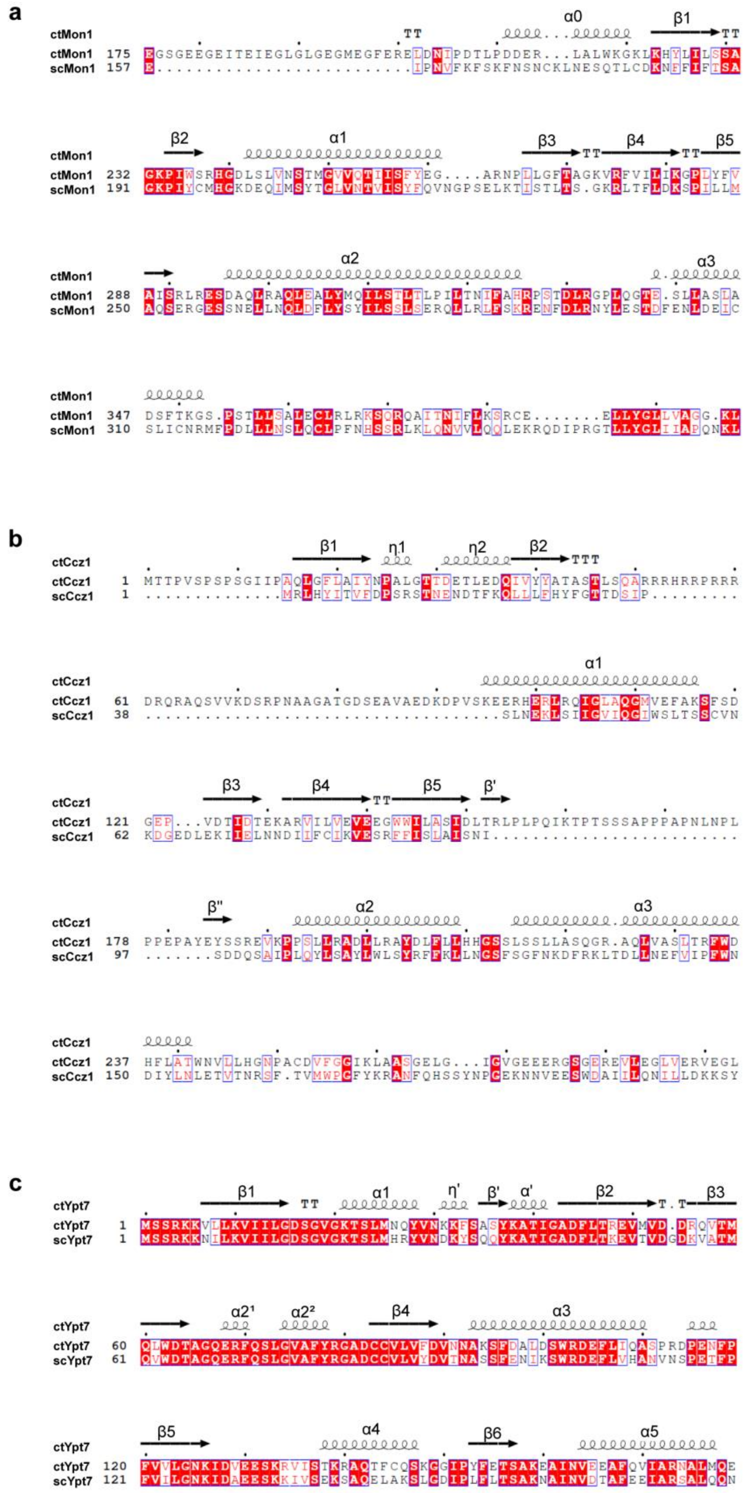

Supplementary Figure 3. A multiple sequence alignment of the (a) Mon1 longin domain, (b) Ccz1 longin domain and (c) of Ypt7 from *C. thermophilum* and *S. cerevisiae* was generated with ClustalW(1) and visualized with ESPript(2). Similar and conserved positions are marked by red font or boxes, respectively. Secondary structure elements as observed in the *Ct*MC1core-Ypt7 crystal structure are labeled and shown above the alignment.

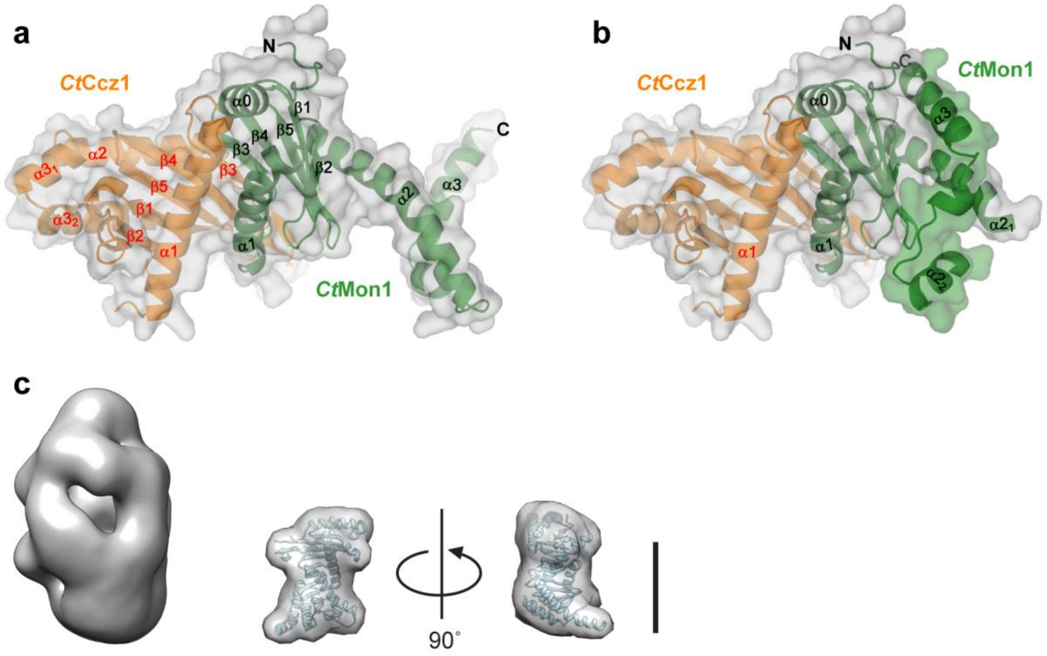

Supplementary Figure 4. Domain swap in the crystal structure of the *Ct*MC1core complex. (a) Structure of the Mon1-Ccz1 heterodimer as observed in the crystal with labeled secondary structure elements. Helix α3 of Mon1 undergoes a domain swap and is swung out to interact with the second copy of Mon1 in the asymmetric unit. (b) Corrected functional unit of the Mon1-Ccz1 heterodimer. Helix α2 of Mon1 is interrupted by proline 317, which is the likely hinge of the domain swap. Thus, the α2-α3 linker region and helix α3 of Mon1 is substituted by the equivalent segment from the second copy of Mon1. (c) Domain swap corrected model of the Mon1-Ccz1 longin heterodimer at 17Å resolution shown in comparison to the negative stain EM reconstruction of the full complex on the left. Scale bar is 5 nm.

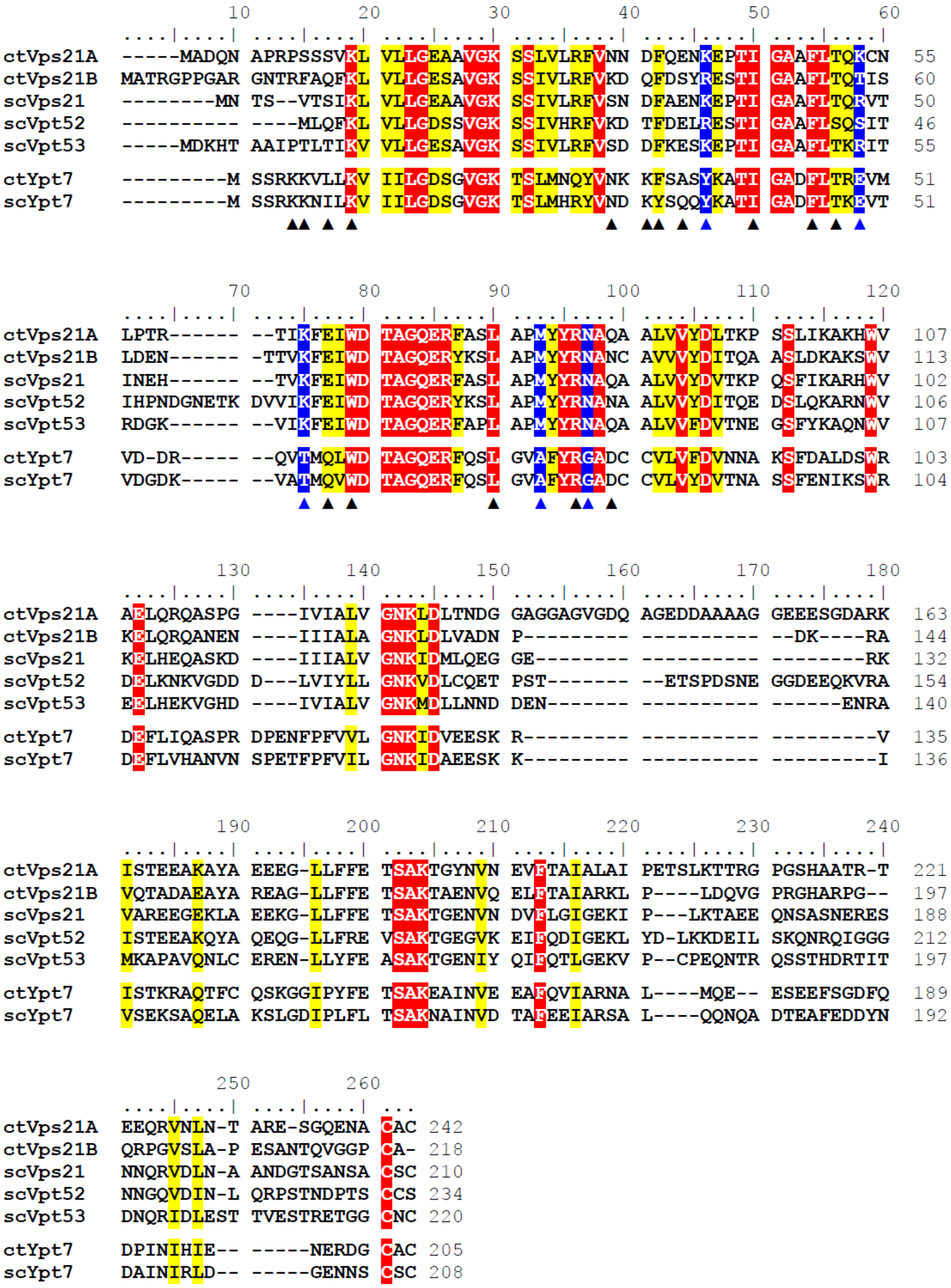

Supplementary Figure 5. Sequence alignment of Vps21 and Ypt7 isoforms from *S. cerevisiae* and *C. thermophilum.* Identical residues are highlighted in red and similar residues in yellow. Residues in Ypt7 that interact with MC1 are marked by triangles (▲). Interacting residues that are conserved for Ypt7 but differ in Vps21 are shaded blue.

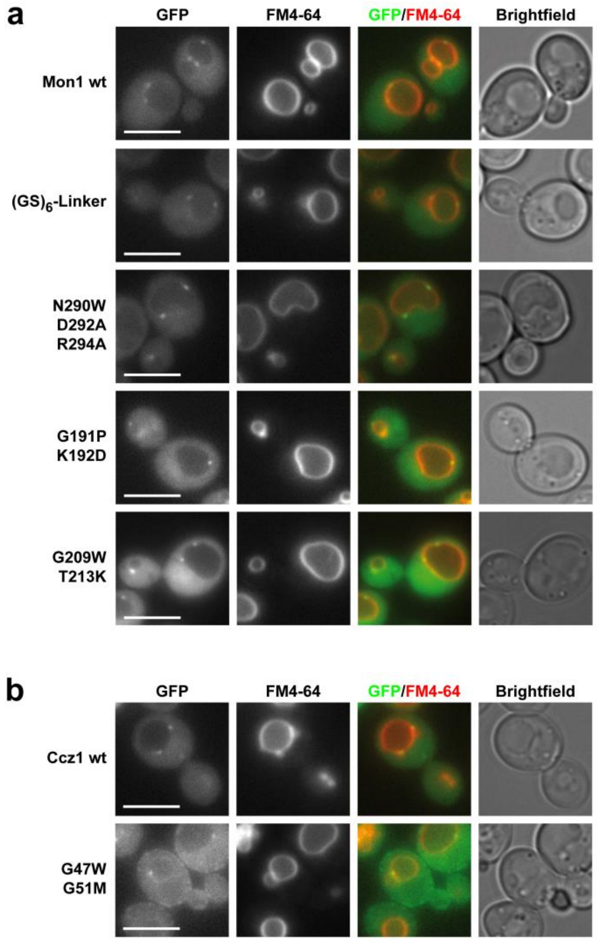

Supplementary Figure 6. Localization of MC1 mutants. GFP-tagged constructs of Mon1 (a) and Ccz1 (b) are expressed in a yeast wild-type background strain. Wild-type and mutant constructs show proper localization to dot-like endosomal structures. Scale bars represent 5 µm.

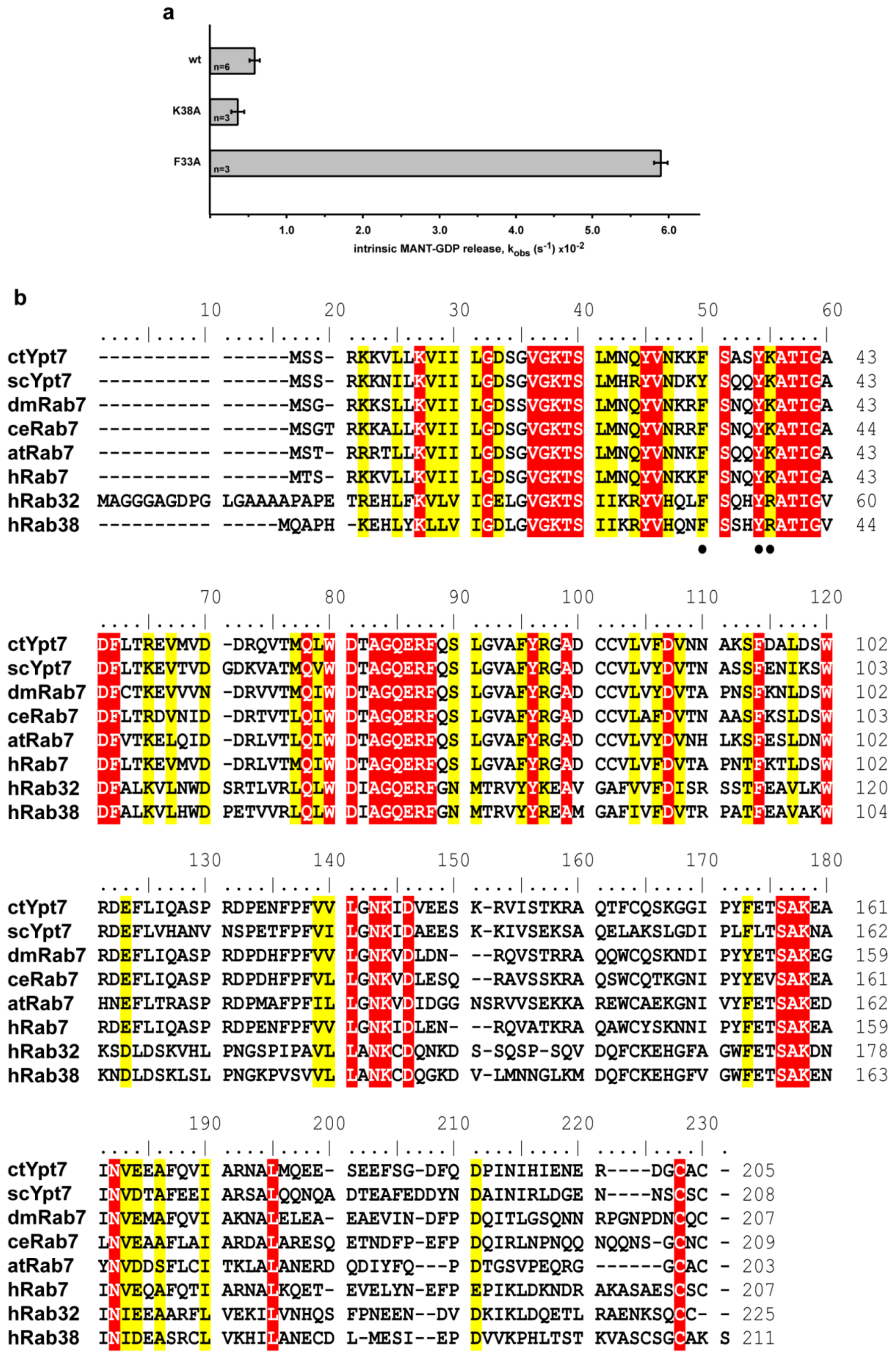

Supplementary Figure 7. (a) Intrinsic nucleotide release rates of *Ct*Ypt7 wild-type protein, K38A and F33A as determined by MANT-GDP dissociation. Data represent the mean of technical repeats with standard deviation. (b) Sequence alignment of Ypt7/Rab7 from different species, human Rab32 and human Rab38. Identical residues are highlighted in red and similar residues in yellow. The position the critical residues F33, Y37 and K38 in *Ct*Ypt7 are marked by a circle (●).

Supplementary Table 1. List of interacting residues between Ypt7 and Mon1-Ccz1.

Ypt7	Mon1	Ccz1
K5	Q336, **E339**	
K6	*R332*	
L8	L242	
K10	*A231*	
N30	*D211*	
K32	***D212***	
F33	D211, L244, *S247*	
A35	S247, V251	
Y37	V251	G110, E113, F114
I41	T254, F285	L107, G110
F45	G250, V251, T254	
T47	*N246*	
E49	S243	
T58	L242, N246	
Q60	G232, N246, *G250*	
W62	A231, G250, Q253, T254	
L73	F258	
A76	*Q253*, S257	
R79	*S230*, Q253	
G80	A231, P327, S328	
D82	***K233***	

Salt bridges and hydrogen bonds are highlighted by bold and italics font, respectively.

Supplementary Table 2. Codon-optimized *Ct*MC1 genes

*Ct*Mon1

GGATCCATGACACAAAATAATGCTCCTGAAAGCGTAGCGGCAGAAGGCACCGTAGCGGGCCAACAGACCGACACCACCAAACCTGAAC
CATCAAGTCAGCCGGCGCCTGAACCACCGTCCCAAGTTACCACTGACTTTCCTCCAAATAACAAGCCGAATGATGTGCCTGCTACTGAACC
AGCATCTTCAAGCCCAAGACCGACAGAACCGCCTGCTCCACCGCCTCCAAAACCTACCATTGCCTTGTCTCCACTGGACATCGCGACACTG
ACGTTTCCTGATGGTACTCGCGGCACATTCCCAGCCCCGCCTCAGACTTCTGCGCAATCAGTCAGCACACCGTCAATTGCAAGCGGTCAT
GTAACTCCTCAGCGCGACACCGATACTGCTAGTATCACGTCCGTTGCAGGTACCCTGCGTGGTGTGACTGGTACGGCTGGTGACTTGGCG
TCACTGTTAGCTGGTGATGGCCTGGGTCGTAAAAGCAAGGCCTGGAGAGTTCTGCGCGCACAACAGCAAGCCTGTGGCGAAGGTTCAG
GCGAAGAAGGTGAAATTACTGAAATCGAAGGTCTGGGCTTAGGTGAAGGCATGGAAGGCTTCGAACGTGAATTAGACAATATTCCGGA
TACATTGCCTGATGACGAAAGATTGGCTCTGTGGAAGGGCAAGCTGAAGCATTACTTGATCTTGAGTTCCGCAGGTAAACCGATCTGGTC
ACGCCACGGCGATCTGTCATTAGTCAATAGCACTATGGGTGTTGTGCAAACAATTATCAGCTTTTATGAAGGCGCCAGAAACCCGTTGCT
GGGTTTTACTGCGGGCAAGGTCCGCTTCGTAATTTTGATCAAAGGTCCTCTGTACTTCGTGGCAATTTCACGCCTGCGTGAAAGCGATGC
GCAGTTAAGAGCTCAATTAGAAGCATTGTATATGCAGATTCTGAGTACCTTGACTCTGCCGATTTTAACGAACATCTTTGCCCACAGACCG
TCCACCGACTTACGCGGTCCTTTGCAAGGCACTGAAAGTTTATTGGCTTCCTTAGCAGATTCGTTCACGAAAGGTAGTCCATCCACCCTGT
TATCGGCCCTGGAATGTTTAAGATTGCGCAAGTCTCAGCGCCAAGCGATTACAAATATCTTTCTGAAATCCCGTTGCGAAGAATTGCTGT
ATGGTTTATTGGTGGCGGGTGGCAAGCTGGTTTCTGTGATTCGTCCAAGAAAACATTCTTTACACCCGTCAGATCTGCAGTTAATCTTTAA
CATGCTGTTCGAATCTGGTGGCATTAAGGGTAATGGTGGCGAAAACTGGATTCCATTGTGCCTGCCGGCTTTTAATAACACCGGCTATTT
GTACATGTATGTTAGTTTCCTGGATGACAAGGCCCCAGATGACCAGAATCAACCACCGGAATCGTCTAACTTGGATGCGAGCAATAAAAA
CTCAAGCAATACTCCTGATGACGATCTGACAGCTTTAATTTTGATCTCGCCATCTCGTGAAGCCTTTTACGCGTTACAGTCTATGCGCACAC
GTTTAGTGAGCCAACTGTTAAGTACGGGTTATCTGTCCTTAATTCGTTCGACCGCACTGTCTGGCAGACCGTCGATTACGTCTATCTTACC
TAAGACCCCATTGCTGCATTTCTTGTACAAATCAAGACCAAACGTCCAATGGTGTATGAGTTCCCTGTCGTCTTTAACCCCTCCAGGTGCC
ACAGCGACGGAAACCTTATTGGCCCGTAGAAAACTGATGAGCGTATACGAAGAACTGCATGCTGCATTACACGCTCGCCATGCACACCT
GCGTGTCGTATATTCGACGGCTGATGAAAAAGAAGGTGAAGGCTTGGCATGCCTGGGTTGGAGTACCCCAGCTTTTGAAGTTTATTGTG
TGGCACCTGGTTGCGTTGGTAGAGCTGGTATGGCGCGTGAAGTCAACAGAGTAGTTCAGTGGGCAAGAAGAGAAGAAGAAAGATTATT
TATTTTGGGTGGTGGTGTGTTTTGAGCGGCCGC

*Ct*Ccz1

GGATCCATGACCACTCCAGTCTCCCCTTCGCCATCAGGCATTATTCCAGCACAGTTGGGTTTCTTAGCAATCTACAATCCAGCGTTGGGCA
CCACTGACGAAACACTGGAAGATCAAATTGTTTATTACGCTACGGCATCGACCTTATCTCAGGCCCGTAGACGCCATCGTAGACCACGCC
GTAGAGACCGCCAACGTGCTCAGTCGGTTGTGAAAGATTCTCGCCCGAATGCTGCAGGTGCAACAGGCGATAGCGAAGCCGTAGCGGA
AGACAAAGATCCAGTTAGTAAGGAAGAACGTCATGAAAGACTGCGCCAAATTGGTTTAGCTCAGGGCATGGTGGAATTTGCAAAAAGTT
TCTCCGATGGTGAACCGGTAGACACTATTGATACAGAAAAGGCCCGTGTTATCCTGGTAGAAGTTGAAGAAGGTTGGTGGATTTTGGCG
TCTATCGATCTGACCAGATTACCGTTGCCTCAAATCAAGACGCCTACCTCTTCAAGCGCTCCACCGCCTGCACCGAATTTGAACCCTCTGCC
ACCGGAACCAGCTTATGAATACAGTTCCCGTGAAGTTAAACCACCATCTCTGTTACGTGCCGACTTGCTGAGAGCTTATGATTTGTTTCTG
TTGCATCACGGCTCGTCTTTGTCAAGCCTGTTAGCAAGTCAAGGTCGCGCCCAGTTGGTGGCGTCCCTGACTCGTTTTTGGGACCATTTCC
TGGCCACATGGAATGTGTTGCTGCACGGTAACCCAGCGTGTGATGTCTTCGGTGGCATTAAATTAGCCGCGTCCGGTGAATTGGGTATC
GGCGTAGGTGAAGAAGAACGCGGCTCTGGTGAACGTGAAGTTTTAGAAGGTTTGGTAGAAAGAGTTGAAGGCCTGGTGGATGTCGTA
GTTGGTCGCTATGGTGGCCCGCCTTCTGAAAAAGGCCCGGAAGAAGAACAATGGCTGGGTTTAGGTGGCGAAGTTGGTGAAGAAGACG
GCGCCGTATTTTTAGGCGTTGGTGCGTTGGATAGAAAATCACTGCGCGGTGTGGTCCAGTGGATGGAAGAAGTGTACGTCTGGGGTGA
AAATGCCTTCGGCGTTGGTGGCTCAAGAGCTGCACGCCGTAGACGCAAAAGAGGTGCTAAGGAAAGCCGCGTGCTGAAAACTGGCGCA
GGTAAAAAGATTCCAATCGGCACTGGTACAGGCACGGAAGGTAAACGTGGTGAAGCAGCAACGCCAGACATGGCAGGTACCCCGGGC
AGTATGGATAAACTGTTTTCCTATTTGACTCTGGGTTACGGCACATCGTGGTCTTTAGGTTTGTCAGGCACCAGCACTCCTTCAGACAGCG
ATCCAGGTAGTGTGTCCAAAGGCATGGTCGAAAGTTCCGAATCGGGTAAACCGCGTAGAGATCTGTCTACGGGTCATTTTCTGTTGGGCT
TGAGTGAATGCTCCGAAGAAGAACTGACTTCGTCTCAAGCTAACCCTAAAGCAATTTTCGTCGAATTAAAGCCGTCATATCAACATCCTAG
CAGAAAAATCCCACCGGAAGACCCACAGCCGCTGGGTAAAGTGGGCCCGGAATTACCTCGCGATCACACAGCACGTCTGAGACCAGTTA
TCTATGTCTCTCAGCCGTTTATCTATATCCTGTTATTCTCAGAAATCACCCCTTCGCCATCTACGTGGCCGACCTTAGCTGAATCGTTGCAT
GCACAACTGTCTCCATTACAGAAACCGTTGCTGCACTCAACCAGCTACCGCCCAGAACGTCCGGTAGTTGAAACCACTTCAAGCAGTGGT
ACAACGACCCAACATCAGATTTTTGACCTGGTTTATGATACAGAAACGCTGACCTTACAAAGTACTATTCCGAATATCCCAGATCCATTCC
CATACTCAGCTACTACACCTACGGGTCATAGCACCGGCCAACAGCATCACCAACAGAGCATTTGGACGCGCGTGGAAGCTTTACAAACCC
ACGCCCAGATTTTGGCGATCCTGTCCTCGGGTCGTGCAATCCCTACTGATCCATCTTCTTTTACCCATCTGCCGTGGGAAGAAGGCGAACG
TACTTGTAAAACAGCAAGAGGTTGGTGGATTGTCTGGACCCGTGTGGTCGAACACTCTCCACCAGATGCCGTAAGCCTGCATCACGCGA
GAGATGACGATGACAACGATGACGATGCCAGTTGCTCCGTGTTAGGCCATTTGCGTTCAGTCAGCAGTTCCCACGCTGCAGGTAGTACTT
CGTCTTCAAGCGGTTCCGGTTTCGGTTTGGGTGCAATTCCTGGTCTGGGTGGCTTAGGTGGCTGGGCAGCAGATGGTGCTACAAGACTG
GCACAGGGTATTGGCATTGACACCAGACGCTATGTGGAAGGCTTGTTGACCTCGTTGGGTAGATAAGCGGCCGC

Supplementary Table 3. List of primers used in this study

Protein construct	Primer
*Ct*Mon1<br>aa 141-665	5’-CGGGC GGATCC ACT GGT ACG GCT GGT GAC TTG-3’<br>5’-CGATT GCGGCCGC TCA AAA CAC ACC ACC ACC C-3’
*Ct*Mon1<br>aa 195-355	5’-GCTCG GGATCC ATG GAA GGC TTC GAA CGT G-3’<br>5’-GAAAT GCGGCCGC TCA GGA TGG ACT ACC TTT CGT G-3’
*Ct*Ccz1<br>aa 1-249	5’-CCATA GGATCC ATG ACC ACT CCA GTC TCC CC-3’<br>5’-GTATT GCGGCCGC TTA GTT ACC GTG CAG CAA CAC-3’
*Ct*Ypt7<br>full length	5’-GGCCG GGATCC ATG TCG TCC AGG AAG AAG G-3’<br>5’-CAAAT GCGGCCGC TCA GCA AGC GCA CCC ATC C-3’
*Ct*Vps21<br>full length	5’-CGTTA GGATCC ATG GCT ACT CGG GGA CCG CCC-3’<br>5’-CAAAT GCGGCCGC TCA GCA AGC GCA AGG CCC GCC AAC-3’
*Sc*Mon1-ΔN<br>aa 158-644	5’-CCTAATGTATTCAAGTTTTCGAAG-3’<br>5’-GGATCTCTTGTACAGCTC-3’
*Sc*Mon1-ΔC<br>aa 158-319	5’-TAACTCGAGGGGGGGCCC-3’<br>5’-ATCCGGGAACATTCTGTTACAGATTAGTG-3’
*Sc*Ccz1-ΔC<br>aa 1-162	5’-TAAGTCGACCTCGAGGGG-3’<br>5’-TGATCTATTCGTTACAGTTTCTAGATTC-3’
*Sc*Mon1-ΔC<br>FYVE	5’-CACTAATCTGTAACAGAATGTTCCCGGATTGGCAATCTAGTCAAC-3’<br>5’-GAATTGGGTACCGGGCCCCCCCTCGAGTTATCCTTGCAAGTCATTG-3’

Mutant	Primer
*Ct*Mon1<br>G232P/K233D	5’-GAGTTCCGCACCTGACCCGATCTGGTCACGC-3’<br>5’-AAGATCAAGTAATGCTTCAG-3’
*Ct*Mon1<br>G250W/T254K	5’-GCAAAAAATTATCAGCTTTTATGAAGGC-3’<br>5’-ACAACCCACATAGTGCTATTGACTAATGAC-3’
*Ct*Mon1<br>S328W/D330A/R332A	5’-CTTAGCCGGTCCTTTGCAAGGCACT-3’<br>5’-GCGGTCCACGGTCTGTGGGCAAAGATG-3’
*Ct*Mon1<br>$(GS)_6$-Linker	5’-GGTTCTGGTTCTGGTAGTGAAAGTTTATTGGCTTCCTTAG-3’<br>5’-AGAACCAGAACCAGAGCCACTCAGAATCTGCATATACAATG-3’
*Ct*Ccz1<br>G106W/G110M	5’-TCAGATGATGGTGGAATTTGCAAAAAG-3’<br>5’-GCTAACCAAATTTGGCGCAGTCTTTC-3’
*Ct*Ypt7<br>F33A	5’-CAACAAGAAGGCCAGCGCTAGCTAC-3’<br>5’-ACATATTGGTTCATCAAACTC-3’
*Ct*Ypt7<br>Y37R	5’-CAGCGCTAGCCGCAAGGCGACTATC-3’<br>5’-AACTTCTTGTTGACATATTGGTTC-3’
*Ct*Ypt7<br>K38A	5’-CGCTAGCTACGCGGCGACTATC-3’<br>5’-CTGAACTTCTTGTTGACATATTG-3’
*Ct*Ypt7<br>E47R	5’-CTTGACTCGGCGCGTCATGGTGGACG-3’<br>5’-AAATCGGCGCCGATAGTC-3’

*Ct*Ypt7<br>T58K	5’-TCGCCAGGTGAAAATGCAGCTCT-3’<br>5’-TCGTCCACCATGACCTCC-3’
*Ct*Ypt7<br>A76M/G80N	5’-CCGCAACGCTGATTGCTGCGTGCTG-3’<br>5’-TAGAACATCACGCCGAGCGACTGGAA-3’
*Ct*Ypt7<br>N125I	5’-GTCGTGCTCGGGATCAAGATCGATGTTGAGG-3’<br>5’-CCTCAACATCGATCTTGATCCCGAGCACGAC-3’
*Sc*Mon1<br>G191P/K192D	Cabrera *et al.* (3)
*Sc*Mon1<br>G209W/T213K	Cabrera *et al* (3).
*Sc*Mon1<br>N290W/D292A/R294A	5’-CCTGGCAAATTATCTAGAAAGCACAGATTTTG-3’<br>5’-GCGAACCATTCTCTTTTGGAAAATAACCTAAG-3’
*Sc*Mon1<br>$(GS)_6$-Linker	5’-GGGTCGGGTTCTGGATCAGATTTTGAAAATTTAGACGAAATATG-3’<br>5’-TGAACCGGAGCCAGATCCCGAAAGAATATACGAATATAAAAAATC-3’
*Ct*Ccz1<br>G47W/G51M	Cabrera *et al.* (3)

Supplementary References

1. Thompson, J. D., Higgins, D. G., and Gibson, T. J. (1994) CLUSTAL W: improving the sensitivity of progressive multiple sequence alignment through sequence weighting, position-specific gap penalties and weight matrix choice. *Nucleic Acids Res.* **22**, 4673–80

2. Robert, X., and Gouet, P. (2014) Deciphering key features in protein structures with the new ENDscript server. *Nucleic Acids Res.* **42**, W320–4

3. Cabrera, M., Nordmann, M., Perz, A., Schmedt, D., Gerondopoulos, A., Barr, F., Piehler, J., Engelbrecht-Vandré, S., and Ungermann, C. (2014) The Mon1-Ccz1 GEF activates the Rab7 GTPase Ypt7 via a longin-fold-Rab interface and association with PI3P-positive membranes. *J. Cell Sci.* **127**, 1043–51
